# Advantages of total parathyroidectomy in patients with secondary hyperparathyroidism induced by end stage renal disease

**DOI:** 10.3389/fendo.2023.1191914

**Published:** 2023-11-22

**Authors:** Cristian Iorga, Cristina Raluca Iorga, Iuliana Andreiana, Iustinian Bengulescu, Traian Constantin, Victor Strambu

**Affiliations:** ^1^ Faculty of Medicine, “Carol Davila” University of Medicine and Pharmacy, Bucharest, Romania; ^2^ Surgery Clinic, “Dr. Carol Davila” Clinical Nephrology Hospital, Bucharest, Romania; ^3^ Nephrology Clinic, “Dr. Carol Davila” Clinical Nephrology Hospital, Bucharest, Romania; ^4^ Department of Urology, “Prof. Dr. Th. Burghele” Hospital, Bucharest, Romania

**Keywords:** secondary hyperparathyroidism, total parathyroidectomy, parathormone, chronic kidney disease, hyperparathyroidism recurrence

## Abstract

**Introduction:**

Secondary hyperparathyroidism, as a result of chronic kidney disease could be treated medically or surgically. When pharmacotherapy fails, patients undergo surgery - parathyroidectomy, the curative treatment of secondary hyperparathyroidism (SHPT). There are currently 3 accepted surgical techniques, each with supporters or opponents – total parathyroidectomy, subtotal parathyroidectomy and parathyroidectomy with immediate autotransplantation.

**Methods:**

In this paper we described our experience on a series of 160 consecutive patients diagnosed with secondary hyperparathyroidism who underwent surgery, in 27 cases it was totalization of the intervention (patients with previously performed subtotal parathyroidectomy or with supernumerary glands and SHPT recurrence). We routinely perform total parathyroidectomy, the method that we believe offers the best results.

**Results:**

The group of patients was studied according to demographic criteria, paraclinical balance, clinical symptomatology, pre- and postoperative iPTH (intact parathormone) values, SHPT recurrence, number of reinterventions. In 31 cases we found gland ectopy and in 15 cases we discovered supernumerary parathyroids. A percentage of 96.24% of patients with total parathyroidectomy did not show recurrence.

**Discussions:**

After analyzing the obtained results, our conclusion was that total parathyroidectomy is the intervention of choice for patients suffering from secondary hyperparathyroidism when pharmacotherapy fails in order to prevent recurrence of the disease and to correct the metabolic parameters.

## 1 Introduction

Secondary hyperparathyroidism is a frequent complication of patients on chronic dialysis. It is characterized by inappropriate secretion of PTH secondary to a phosphocalcic metabolic disorder. This will affect several organs and systems: bone (causing demineralization, pain, fractures), cardiovascular (causing calcifications of the arterial walls, myocardial wall, heart valves), calcifications in viscera, periarticular tissue, skin, eye. When looking at increased morbidity and mortality the most important complications are the cardiovascular progressive calcifications which will lead to myocardial angina or infarction, high blood pressure, cardiac failure, calciphylaxis. Other manifestations include psychological, neurological disorders and malnutrition. These complications aggravate the prognosis of patients with secondary hyperparathyroidism and also increase the surgical anesthetic risk (1, 2).

Patients with end stage renal disease under dialysis (hemodialysis or peritoneal dialysis) have low levels of 1,25 (OH) 2 D3, which results in a decrease in calcium absorption which leads to hypocalcemia and a secondary increase in PTH secretion. This is the trigger that will lead to secondary hyperparathyroidism (3). The occurrence of secondary hyperparathyroidism is based on several factors - vitamin D deficiency, phosphorus retention, decreased calcium receptor activity in parathyroids. When the renal function decreases, phosphorus excretion decreases thus leading to its increased level in the blood, hypocalcemia and decreased vitamin D levels. The decrease of the plasma level of vitamin D will also affect the intestinal calcium absorption. All these factors and mechanisms will ultimately result in hypocalcemia and stimulate the increased secretion of PTH. Lately, it is considered that fibroblast growth factor 23 (FGF-23), which increases in kidney failure probably due to increased plasma phosphorus levels, may be responsible for decreasing vitamin D synthesis (4).

The current consensus for PTH values in dialyzed patients, with or without medical treatment for secondary hyperparathyroidism is that it should be maintained between 2x-9x times higher than the reference range (usually accepted at 130-600pg/ml). To ensure that PTH values are maintained in this range, it is recommended to monitor its level every 3-6 months. If changes occur outside of the reference range, immediate action must be taken either by changing the medical treatment or by referring the patient to surgery (5, 6).

The World Health Organization through the Expert Committee on Biological Standardization reported the preparation of a recombinant human PTH 1-84 to serve as the international reference standard. It is called 95/646. Even with this standard, it is recognized that laboratories have their own normal values and it is recommended that they inform physicians about the methods of determination used to make the biochemical interpretation as correct as possible (7).

It is well known that PTH is an unstable hormone when measured by standard lab techniques its stability being dependent on processing (plasma or serum), way of sampling (on heparin or EDTA), storage mode until processing, but also on the processing method, calibration of equipment and, last but not least, the reproducibility of the result. Parathyroid hormone has a polypeptide structure, containing a chain of 84 amino acids. It is an unstable molecule that is quickly removed from bloodstream, being metabolized and cleaved into fragments. The standard measurement of parathyroid hormone is intact PTH (iPTH). The iPTH assay reacts not only with 1-84 PTH but also with truncated fragments (metabolic active) such as 7-84 PTH. However, the PTH remains the main biochemical marker used to monitor the progression of patients with secondary hyperparathyroidism. With the help of the new 95/646 PTH standard, new advances are made to improve mass spectrometry which will result in comparable PTH results. This demonstrates the concern to obtain accurate and reproducible results in any laboratory tests. As of today we already benefit from the third generation of immunological tests for PTH (8–10).

Therapy for these patients is primarily pharmacologic: calcimimetics, vitamin D analogues and phosphate binders. Treatment should be conducted along with monitoring the serum parameters: calcemia, phosphatemia, alkaline phosphatase levels. Pharmacotherapy is considered acceptable as long as the values of calcium and phosphate concentrations and PTH are maintained “normal”: serum calcium 8.6-10.5 mg/dl, serum phosphate 2.5-4.5mg/dl,iPTH ≤100-300 pg/ml (2, 3). The treatment aims to prevent the complications of renal osteodystrophy, the most important being represented by the complications of the cardiovascular system, as mentioned above (11). These cardiovascular complications through the deposition of calcium in the intima and vascular media increase the risk of death from cardiovascular diseases and considerably affect the viability of vascular access (long life catheter or arterio-venous fistula (12).

With all advances in pharmacotherapy for secondary hyperparathyroidism, at some point, the disease will become refractory to treatment, and surgical intervention is the only curative solution (13). Total parathyroidectomy is indicated in 20% of patients with chronic renal failure that have been on dialysis between 3-10 years, and the percentage is doubled when dialysis length reaches 20 years.

The purpose of this paper is to perform an analysis of the secondary hyperparathyroidism recurrence incidence depending on the operative technique. We also want to analyze the factors that cause the recurrence of the disease.

## 2 Materials and methods

A group of 165 consecutive patients admitted to the General Surgery Clinic of “Dr. Carol Davila” Clinical Hospital of Nephrology for primary and secondary hyperparathyroidism was analyzed. The study was performed retrospectively and prospectively and all the statistical data were entered into an MS Office-Excel database.

From this group, 160 patients were admitted with the diagnosis of secondary hyperparathyroidism, all patients with stage 5 of renal failure on hemodialysis or peritoneal dialysis.

The group was analyzed based on age, gender, pre and postoperative iPTH values, preoperative alkaline phosphatase values, pre and post-operative calcium values, clinical symptomatology, pre and postoperative imaging investigations, associated comorbidities, duration of dialysis period, type of surgical intervention, histopathological form of excised parathyroids.

Out of these patients 27 were reoperated due to the recurrence of secondary hyperparathyroidism, 5 of which were primarily operated in our clinic, the remaining 22 were referred from other nephrology services for the same diagnosis.

## 3 Results

Gender wise the subjects were almost equally divided by 77 females and 83 males. The average age was 51.75 years old with extremes between 25 and 72 years.

Of the 160 patients with secondary hyperparathyroidism 12 were undergoing peritoneal dialysis and the remaining 148 were on hemodialysis. Patients on peritoneal dialysis had been only on this type of dialysis and had a median dialysis period of 7.25 years with extremes between 5 and 15 years. Patients on hemodialysis had a median dialysis period of 8.35 years with extremes ranging from 5 to 20 years. Most patients on hemodialysis N = 71 were in dialysis between 9 and 14 years. Of the patients with peritoneal dialysis half of them were on dialysis between 5 and 8 years (**Table 1**).

**Table 1:** Lot structure.

	Gender		Type of dialysis		Type of surgery	
	Female	Male	Hemo	Peritoneal	Primary	Recurrence
Number	77	83	148	12	133	27
Percentage	48%	52%	92,5%	7,5%	83,1%	16,9%
Total	160		160		160	

**Figure 1:**
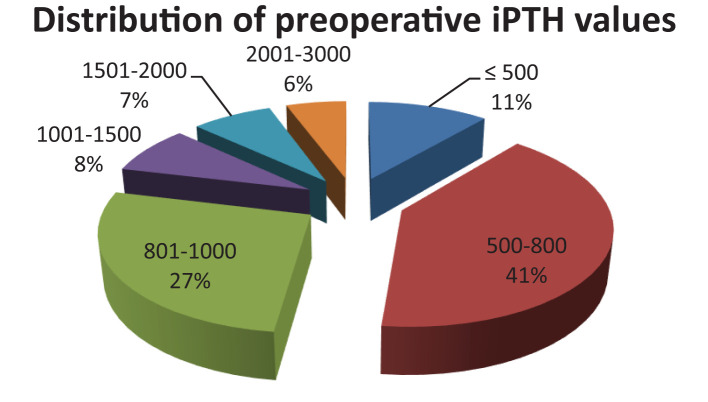
Distribution of preoperative iPTH values.

All of the 160 patients had increased preoperative iPTH values, 68% having values ranging from 500-1000 pg/ml (**Figure 1**) with extreme values ranging between 325-2275pg/ml.

Even if guidelines recommend surgical intervention at iPTH values above 500 pg/ml, 11% of our cases had iPTH ranging from 325-500 pg/ml. These specific cases included patients with recurrence of the disease because of known subtotal parathyroidectomy (e.g. not being able to identify four parathyroid glands, pathological examination that did not identify parathyroid tissue), with supernumerary parathyroids and with persistent clinical symptoms.

When analyzing the preoperative iPTH values between the two groups receiving dialysis (peritoneal or hemodialysis) it was observed that the preoperative values of iPTH, while maintaining the same reference interval as above, were recorded in percentages of equal sensitivity. It should be mentioned that the two groups have very different numerical values (12 patients with peritoneal dialysis and 148 patients with hemodialysis).

Analysis of calcium values distribution before surgery showed that most patients, 43%, had values ranging from 11.6-12.5 mg/dl with extremes between 10.6 and 14.3 mg/dl. The alkaline phosphatase values were increased in all patients with extreme values between 135-1060UI/l.

In terms of imaging investigations, there is no statistical consistency in the group:36 patients underwent parathyroid ultrasound, 47 patients had parathyroid scintigraphy with 99Tc-sestamibi and 38 patients benefited from cervical and mediastinal computed tomography. The rest of the patients did not have any preoperative imaging investigations.

The whole group of patients was subjected to surgery. Surgical intervention was total parathyroidectomy without reimplantation in the sternocleidomastoid muscle or forearm, none of the patients being a candidate for renal transplantation. Reintervention was performed on 27 patients suffering from recurrence of hyperparathyroidism, 5 of the recurrent patients were primarily operated in our clinic and the recurrence of hyperparathyroidism occurred within a range of 5 to 9 months. The same time period of hyperparathyroidism recurrence was also seen in the other 22 patients. The recurrence rate in the group of patients operated in our clinic was 3.76%. 2 of the 5 patients with disease recurrence had subtotal parathyroidectomy due to the fact that one of the parathyroids could not be identified intraoperatively (these patients did not have specific imaging investigations), the other 3 having supernumerary glands. In the cases of the other 22 that were not primary operated in our clinic recurrence was due to subtotal parathyroidectomy as initial surgical indication (11 patients), failure to identify the parathyroid gland (5 patients), supernumerary glands (6 patients). The result of the surgery was confirmed by the histopathological result and iPTH postoperative sampling (**Table 2**).

**Table 2:** Causes of recurrence.

Reffered center	No.	Failure to identify gland	Supernumerary gland	Subtotal parathyroidectomy	Total
Our center	No.	2	3	0	5
Other center	No.	5	6	11	22
Total	No.	7	9	11	27

iPTH was postoperatively sampled in only 70% of the patients group. In the majority of patients, the decrease in iPTH value was major (extreme values between 4.5-98pg/ml) with the exception of the 5 patients who subsequently had the recurrence of hyperparathyroidism. Postoperative iPTH values in these 5 patients were between 255-670pg/ml.

The incidence of parathyroid ectopia in the entire group was 19.37% (N = 31): retrotracheal N = 3, N = 6 intra-thyroid, N = 3 carotid bifurcation, N = 13 tireotimic ligament, tracheoesophageal channel N = 6 (**Table 3**).

**Table 3:** Position of ectopic glands.

Place	Tireotimc ligament	Intrathyroid	Tracheoesophageal fold	Retrotracheal	Carotid bifurcation	Total
No.	13	6	6	3	3	31

Supernumerary glands were found in 15 patients representing 9.37% of the study group.

## 4 Discussion

The last decade has made important contributions to the diagnosis and treatment of secondary hyperparathyroidism. Disease should be diagnosed early and pharmacotherapy promptly instituted. The goal of the treatment should be to reduce iPTH values and maintain a balance of calcium, phosphorus and vitamin D levels. At the moment there are treatment guidelines established by NKF/KDOQI or KDIGO. Medical treatment manages to keep the disease under control, but there are patients who stop treatment, and patients who become refractory to treatment (14).

At this time, the only treatment that can lead to the restoration of biorhythmic is the surgical treatment.

The surgical treatment for patients with secondary hyperparathyroidism consists of subtotal parathyroidectomy, total parathyroidectomy with or without re-implantation/cryopreservation. These surgical interventions can be done through the classic open procedure and with mini-invasive techniques.

To prevent recurrence, we have approached the entire group of patients with total parathyroidectomy. This fact was also confirmed by the recurrence of the disease in all patients in the group that did not succeed in total parathyroidectomy. Also, 96.24% of patients who had total parathyroidectomy confirmed post-operatively by iPTH values and histopathological result, did not subsequently experience recurrence of the disease. The follow-up period of these patients ranged from 3 to 30 months.

In the studied group, the recurrence of hyperparathyroidism was due either to the retention of a parathyroid gland by not identifying it intraoperatively or due to the existence of supernumerary glands. In one case the histopathological result identified that one of the glands alleged to be parathyroid was actually a lymph node. Recent studies confirm the presence of supernumerary glands in a percentage of 5-30%, and ectopic glands in a percentage of 28-46% (15).

We followed the patients postoperatively and observed that iPTH values began to rise and clinical symptomatology reappeared at an interval between 5 to 9 months postoperatively, these cases requiring surgical intervention to suppress the remaining gland. All patients requiring parathyroidectomy aggregation benefited from a preoperative imaging protocol necessary to identify the anatomical position of the remaining gland. This protocol consisted of thyroid ultrasound, scintigraphy with 99Tc-sestamibi and computed tomography of the cervical and mediastinal region (16).

**Figure 2:**
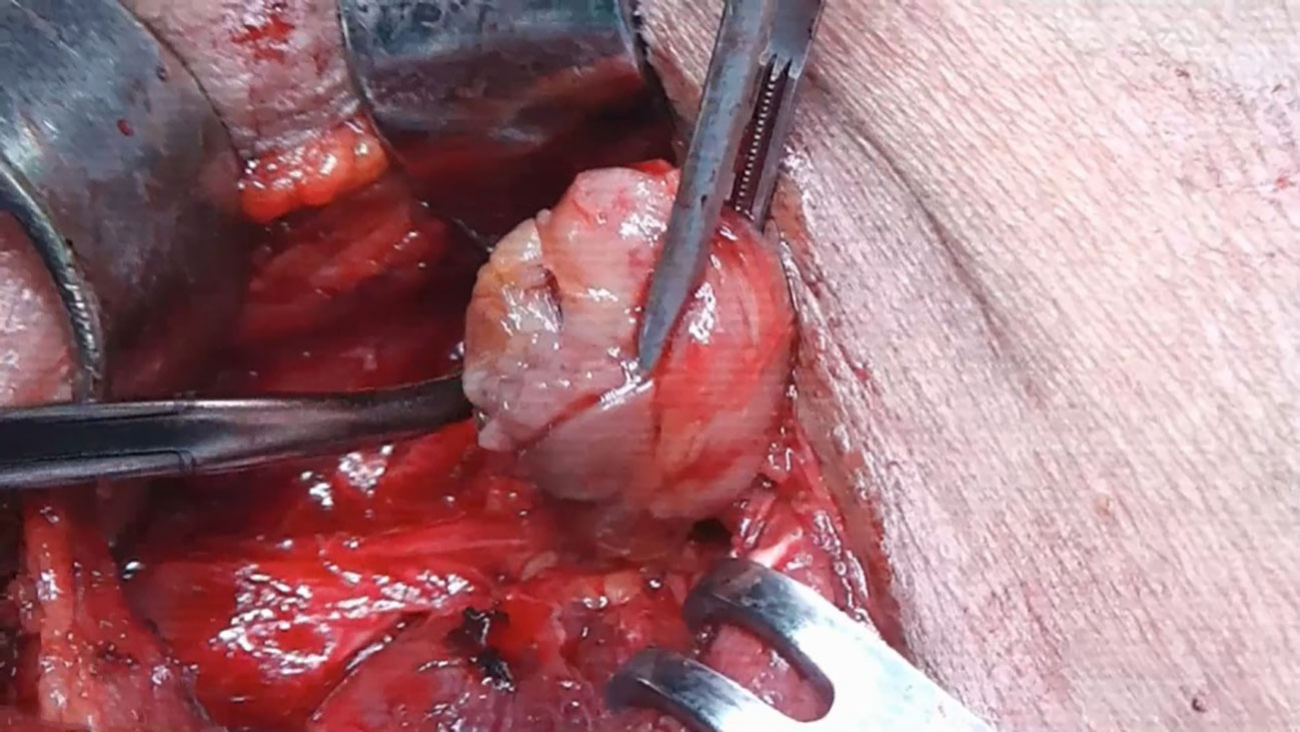
Mediastinal parathyroid adenoma.

Re-intervention led to the identification and surgical excision of the remaining gland. In this case, the result of surgery was confirmed by the histopathological result and the normal values of iPTH (**Figure 2**).

Most authors and the latest consensus for the treatment of secondary hyperparathyroidism support indications for surgical treatment. Although there is still discussion about iPTH values (above 500pg/ml or 800pg/ml) for surgical indication, these will also be clarified in the future by new methods of standardization of iPTH dosing (17, 18).

Every year, approximately 2.5% of patients on dialysis with secondary hyperparathyroidism reach surgery. There are still numerous discussions on the surgical technique adopted. This is due to the increased postoperative morbidity and the recurrence of the disease (19, 20).

On the other hand studies have begun to appear that demonstrates that total parathyroidectomy, in addition to decreasing the risk of recurrence, also decreases mortality due to vascular or other causes. In a metaanalysis performed by Chen et al. a 28% decrease in general mortality and a 37% decrease in cardiovascular disease mortality was identified compared to medically treated patients (21).

The recurrence is considered to be between 15-30% after subtotal or total parathyroidectomy with reimplantation. Some authors consider that there is no significant difference in disease recurrence depending on the two types of surgery: subtotal or total parathyroidectomy with reimplantation. In both cases the recurrence of the disease was due, in particular, to the remaining or transplanted gland (66% of cases) and more rarely due to ectopic or supernumerary glands (22–25). Recently several studies suggest as the first line of action total parathyroidectomy for patients with hyperparathyroidism induced by end stage renal disease. This has the advantage of preventing disease recurrence, preventing associate complications (metabolic, vascular, bone, etc.) and avoiding surgical re-interventions in patients with high surgical and anesthetic risk (26, 27).

There are still many discussions in the literature about the optimal surgical technique for patients who require surgery for secondary hyperparathyroidism. A meta-analysis performed by Liu et al. underlines the fact that at this moment there are not clinical guidelines for the ideal surgical technique used for these patients. In conclusion the meta-analysis shows the superiority of total parathyroidectomy compared to total parathyroidectomy with reimplantation (28).

## 5 Conclusions

Total parathyroidectomy for dialysis patients who can no longer benefit from renal transplantation is a safe intervention that leads to a decrease in the recurrence of secondary hyperparathyroidism and also a decrease in morbidity and mortality. Thorough dissection of the cervical region and correct identification of ectopic or supernumerary glands must be performed in any type of surgical intervention. Preoperative imaging investigations becomes mandatory in the case of disease recurrence. The difficulty in achieving re-interventions in the case of disease recurrence is another argument that indicates total parathyroidectomy as an intervention of choice in secondary hyperparathyroidism.

## Data availability statement

The original contributions presented in the study are included in the article/supplementary material. Further inquiries can be directed to the corresponding author.

## Ethics statement

Ethical approval was not required for the study involving humans in accordance with the local legislation and institutional requirements. Written informed consent to participate in this study was not required from the participants or the participants’ legal guardians/next of kin in accordance with the national legislation and the institutional requirements.

## Author contributions

Concept and study design: CI and CRI. Data collection: IA and IB. Analysis: CRI and VS. Manuscript writing: CRI. Final approval and review: CI, TC, and VS. All authors contributed to the article and approved the submitted version.
